# Opposing roles for 53BP1 during homologous recombination

**DOI:** 10.1093/nar/gkt729

**Published:** 2013-08-22

**Authors:** Andreas Kakarougkas, Amani Ismail, Karolin Klement, Aaron A. Goodarzi, Sandro Conrad, Raimundo Freire, Atsushi Shibata, Markus Lobrich, Penny A. Jeggo

**Affiliations:** ^1^DNA Double Strand Break Repair Laboratory, University of Sussex, Brighton BN1 9RQ, UK, ^2^Department of Biochemistry and Molecular Biology, Southern Alberta Cancer Research Institute, University of Calgary, Alberta T2N 4N1, Canada, ^3^Radiation Biology and DNA Repair Laboratory, Darmstadt University of Technology, 64287 Darmstadt, Germany and ^4^Unidad de Investigación, Hospital Universitario de Canarias, Instituto de Tecnologías Biomédicas, Ofra s/n, 38320 La Laguna, Tenerife, Spain

## Abstract

Although DNA non-homologous end-joining repairs most DNA double-strand breaks (DSBs) in G2 phase, late repairing DSBs undergo resection and repair by homologous recombination (HR). Based on parallels to the situation in G1 cells, previous work has suggested that DSBs that undergo repair by HR predominantly localize to regions of heterochromatin (HC). By using H3K9me3 and H4K20me3 to identify HC regions, we substantiate and extend previous evidence, suggesting that HC-DSBs undergo repair by HR. Next, we examine roles for 53BP1 and BRCA1 in this process. Previous studies have shown that 53BP1 is pro-non-homologous end-joining and anti-HR. Surprisingly, we demonstrate that in G2 phase, 53BP1 is required for HR at HC-DSBs with its role being to promote phosphorylated KAP-1 foci formation. BRCA1, in contrast, is dispensable for pKAP-1 foci formation but relieves the barrier caused by 53BP1. As 53BP1 is retained at irradiation-induced foci during HR, we propose that BRCA1 promotes displacement but retention of 53BP1 to allow resection and any necessary HC modifications to complete HR. In contrast to this role for 53BP1 in HR in G2 phase, we show that it is dispensable for HR in S phase, where HC regions are likely relaxed during replication.

## INTRODUCTION

Agents such as ionizing radiation (IR) generate two-ended DNA double-strand breaks (DSBs) in all cell cycle phases. Additionally, one-ended DSBs can arise in S phase at stalled/collapsed replication forks (1). Cells are equipped with two DSB repair mechanisms, DNA non-homologous end-joining (NHEJ), which occurs in all cell cycle phases and homologous recombination (HR), which uses sister homologues in late S/G2 phase (2,3). NHEJ represents the major pathway repairing two-ended DSBs, whereas HR exerts its main function during replication (4,5).

DSB repair is influenced by chromatin structure and cell cycle phase. In G0/G1, the majority (∼85%) of IR-induced DSBs are located in euchromatic (EC) DNA and are rejoined by NHEJ without requirement for ATM or DDR mediator proteins (6). In contrast, the repair of DSBs located in heterochromatic (HC) regions (∼15%) requires ataxia telangiectasia mutated (ATM), H2AX, MRN, ring finger containing nuclear factor 8 (RNF8), RNF168 and p53 binding protein 1 (53BP1) as well as NHEJ proteins (7,8). Current evidence suggests that compacted HC impedes DSB repair, and that ATM promotes phosphorylation of KRAB domain associated protein 1 (KAP-1) (pKAP-1), an HC-building factor. pKAP-1 forms in a pan-nuclear manner and as discrete pKAP-1 foci. Although pan nuclear pKAP-1 only requires activated ATM, pKAP-1 foci, which form uniquely at HC-DSBs, additionally require 53BP1 and upstream DDR proteins necessary for 53BP1 recruitment (8). 53BP1 is proposed to tether ATM at DSBs promoting concentrated pKAP-1 (i.e. pKAP-1 foci) at HC-DSBs, release of the large isoform of the chromatin remodelling protein, CHD3, and HC relaxation (8,9). Although ATM localizes to all DSBs, it is specifically required for HC-DSB repair (although it may also promote repair of other DSB sub-fractions such as those undergoing transcription) (10). In addition to these differing genetic requirements for HC versus EC-DSB repair, there are kinetic differences; EC-DSBs are repaired rapidly, whereas HC-DSBs are repaired with slow kinetics (7).

In G2, EC-DSBs are repaired predominantly by NHEJ (as in G1). However, HC-DSBs, in contrast to G1, undergo repair by HR (4). ATM has at least two functions in HR; it phosphorylates KAP-1 promoting HC relaxation and phosphorylates and activates CtIP, enabling DNA resection (11). Based on these and additional findings, the emerging model regulating pathway choice is that NHEJ initially attempts to repair DSBs in G2 but if rapid repair does not ensue then resection occurs committing to HR. Thus, HR functions predominantly to repair the slow component of DSB repair, proposed to represent HC-DSBs in G2 phase (4).

53BP1 and BRCA1 are also important in regulating DSB repair pathway choice (11–14). BRCA1, which is essential for HR, has been reported to enhance CtIP recruitment and resection (15–17). 53BP1, in contrast, has been argued to promote NHEJ. Although 53BP1 is dispensable for most DSB repair by NHEJ, it is required for HC-DSB repair in G0/G1 cells, telomere fusions and long range V(D)J recombination rejoining (8,18,19). Importantly, although deficiency in BRCA1 impairs resection and inhibits HR, both are regained following concomitant loss of BRCA1 and 53BP1 in S phase cells (12,13). Thus, it has been proposed that BRCA1 overcomes the barrier to HR posed by 53BP1. A recent study proposed that BRCA1 achieves this by excluding 53BP1 to the irradiation-induced foci (IRIF) periphery, thereby overcoming the inhibitory barrier of 53BP1 on HR (20). However, these findings generate a confliction: the necessity for 53BP1 to relax HC and the suggestion that HC-DSBs undergo repair by HR would necessitate that 53BP1 can promote HR. Yet, other studies suggest rather that 53BP1 solely promotes NHEJ.

Here, we examine the requirement for 53BP1 in HR at IR-induced DSBs in G2. We exploit G2 phase analysis, as it specifically involves the analysis of two-ended DSBs and also allows roles for these proteins in HC-relaxation to be examined. We show that, contrary to previous reports, 53BP1 has a pro-HR function at HC-DSBs in G2 by promoting pKAP-1 foci formation. Thus, 53BP1 does not solely promote NHEJ. 53BP1, however, is dispensable for HR repair of methyl methanesulfonate (MMS) or hydroxyurea (HU)-induced lesions in S phase and for the increased HR that arises at DSBs when NHEJ is compromised. Thus, 53BP1 is not an essential HR protein but can promote HR at HC-DSBs. These findings provide further insight into the interplay between NHEJ and HR in DSB repair and provide an explanation for why 53BP1 is retained at DSBs undergoing HR in G2 phase.

## MATERIALS AND METHODS

### Cell culture and irradiation

A549, U20S, 1Br hTERT, AT1Br hTERT, 2BN hTERT and mouse embryo fibroblasts (MEFs) cells were cultured in Dulbecco’s modified Eagle’s medium with 10% foetal calf serum (FCS), l-glutamine, penicillin and streptomycin at 37°C in a humidified 95% air and 5% CO2 atmosphere. Cells were irradiated by exposure to a ^137^Cs source. For G2 experiments, 3 μg/ml aphidicolin, was added before IR to prevent progression of S-phase cells into G2. ATM inhibitor (ATMi) (Ku-55933) (Calbiochem) was added as indicated. Unless otherwise stated, all results represent the mean and s.d. of three experiments.

### Small interfering RNA knockdown conditions

Small interfering RNA (siRNA)-mediated knockdown was achieved using HiPerFect Transfection Reagent (Qiagen, Hilden, Germany) following the manufacturer’s instructions. siRNA duplexes per 4 × 10^5^ of logarithmically growing cells were used at a final concentration of 20 nM. Cells were then grown for 72 h before IR. 53BP1 (5′-AGAACGAGGAGACGGUAAUAGUGGG-3′), KAP-1 (5′CAGUGCUGCACUAGCUGUGAGGAUA-3′), BRCA1 (5′-GGAACCUGUCUCCACAAAG-3′) and POH1(5′- AGAGUUGGAUGGAAGGUUU-3′) siRNA oligonucleotides were StealthTM RNAi oligos from Invitrogen. The 53BP1, XLF, BRCA1, BRCA2, Ku80 and DNAPK-cs siRNA oligonucleotides were obtained from the Dharmacon SMARTpool (see Supplementary Figure S5 for full list of oligo sequences). The generation of the siRNA resistant pCMH6K-53BP1 constructs used for complementation experiments was described previously (8).

### Immunofluorescence

Cells plated on glass slides were fixed for 10 min with fixative [3% (w/v) Paraformaldehyde (PFA), 2% (w/v) sucrose, 1X phosphate buffered saline (PBS)] and permeabilized for 1 min with 0.2% Triton X-100 in PBS. When staining for RPA and RAD51 foci, pre-extraction was performed by treatment with 0.2% Triton X-100 in PBS for 0.5–1 min before PFA fixation. Cells were rinsed with PBS and incubated with primary antibody diluted in PBS + 2% (w/v) bovine serum albumin (BSA) for 1 h at room temperature (RT). Cells were washed three times, incubated with secondary antibody [diluted in PBS + 2% (w/v) BSA] for 30 min at RT in the dark, incubated with 4′,6-diamidino-2-phenylindole for 10 min and washed three times with PBS. Slides were mounted using Vectasheild and visualised/analysed using a Nikon-e400 microscope and imaged using an Applied Precision® Delta Vision® RT Olympus IX70 deconvolution microscope and softWoRx® Suite software. In each sample, a minimum of 30 cells was scored blindly, and error bars represent the s.d between three experiments. Co-localization analysis (Supplementary Figure 2E) was undertaken using softWoRx® Suite software. The Pearson Coefficient of Correlation indicates the degree of colocalisation between two intensities on a pixel-by-pixel basis (full colocalisation is 1.0).

### Analysis of RPA foci overlap with HC in G2

The 1BR3 hTERT (WT) and 2BN hTERT (XLF mutated) cells were irradiated with 3 Gy IR and maintained in aphidicolin (3 μg/ml). Cells were harvested 8 h later, extracted for 30 s with 0.2% triton X100 (in PBS), fixed and immunostained for RPA (p34 subunit, RPA2) and Histone H3 Trimethylated K9 (H3K9me3) or Histone H4 Trimethylated K20 (H4K20me3). Highly resolved Z-stacks were captured using a Confocal Zeiss LSM510meta microscope. Subsequently, 3D rendering was performed to convert the 3D Z-stacks into a 2D image. The overlap between RPA foci and HC markers was quantified by ImageJ software with the ‘Colocalisation Analysis’ plugin. Mean values represent 15–20 cells in each of three experiments for each condition. See Supplementary Figure S3 for further details of analysis.

### Sister chromatid exchanges and chromosomal breaks

The 2 × 10^5^ logarithmically cells were grown for 48 h in 10 µM BrdU before IR. In all, 0.2 µg/ml Colcemid (plus 1 mM caffeine to overcome the G2/M checkpoint) was added from 8 to 12 h post-IR to collect mitotic cells. For the MMS experiments, cells were treated with 0.5 mM MMS for 0.5 h. MMS was then was washed away with medium; 10 μM EdU was added immediately after MMS treatment to identify cells in S phase at the time of MMS treatment. Caffeine (1 mM) and colcemid (200 ng/ml) were added 9 h post-treatment, and metaphases were harvested 3 h later. For the HU experiments, 1 mM HU was added for 16 h during the second round of DNA duplication in the presence of BrdU. The cells were then washed and incubated for a further 8 h in BrdU-containing media. Caffeine (1 mM) and colcemid (200 ng/ml) were added 3 h later. Sister chromatid exchanges (SCEs) and chromosomal breaks were scored in 20–40 metaphase spreads from three experiments per data point. Staining was according to standard protocols. Aphidicolin (Sigma-Aldrich, Poole, UK) was added at 1 µg/ml immediately before IR. ATM inhibitor (KU55933, Calbiochem) was added at 20 µM 20 min before IR.

### Antibodies

The primary antibodies used were as follows: γH2AX and 53BP1 (Upstate Technology, Billerica, USA) at 1:800, 53BP1 (Bethyl, Cambridge, England) at 1:800, RPA (Lifespan Biosciences, Suffolk, UK) at 1:100, Phospho RPA32 (S4/S8) (Bethyl, Cambridge, UK), Ku80 (Cell signalling), RAD51 (Santa-Cruz Biotechnology, Santa Cruz, USA) at 1:200, H3K9me3, H4K8ac and H4K20me3 (Abcam, Cambridge, UK), at 1:800). The anti-rabbit polyclonal BRCA1 antibody was raised in rabbits injected with human BRCA1 amino acid residues 1350–1650. The corresponding cDNA fragment was cloned into the pET-28 expression vector (Novagen, Billerica, USA) to allow expression of a histidine tagged fusion protein in *Escherichia coli*. Once expressed, the proteins were purified using Ni-NTA resin (Qiagen, Hilden, Germany) following the manufacturer’s instructions and were inocculated into rabbits to raise antibodies using standard procedures (21). The following secondary antibodies were used: FITC (Sigma Aldrich, Poole, UK.) at 1:200, CY3 (Sigma Aldrich, Poole, UK.) at 1:200, Alexa 488, Alexa647 and Alexa 555 (Invitrogen, Grand Island, USA) all at 1:400.

### Analysis of DSB repair following chromatin immunoprecipitation

Hela cells were treated with either scrambled (siMOCK) or BRCA2 siRNA (siBRCA2) and subjected to double thymidine blockage and release. Preliminary IF experiments determined that, by 7 h, cells were enriched (>85%) for pS10-H3 positive cells indicative of G2/M (Supplementary Figure S4A). G2-enriched cells were treated with ±10 Gy IR and harvested 0.5 or 8 h later. Cells were extracted in nucleosome preparation buffer (NPB) containing 100 U/ml micrococcal nuclease, 0.5 μM Microcystin-LR, 1 μM Wortmannin, 1X protease inhibitors (Sigma) and 20 mM N-ethylmaleimide and incubated at 37^°^C for 45 min before being further solubilised by the addition of an equal volume of nucleosome solubilization buffer (NSB), sonicated and clarified by centrifugation (10 000 rpm, 10 min); using this procedure, >99% of chromatin is digested into soluble mono- or di-nucleosomes. One microgram of extract was incubated with 1 µg of ChIP-grade anti-H3K9me3, anti-H4K8ac (Abcam) or pre-immune IgG (=Mock IP) for 2.5 h before addition of 25 µl 1:1 protein-A sepharose beads in lysis buffer (1:1 NPB and NSB) for an additional 0.5 h. Details of the NPB and NSB buffers are described in reference (8). Protein-A sepharose beads were then washed three times with 0.5 ml of lysis buffer and boiled in SDS sample buffer. IP samples (alongside 50 µg of input) were immunoblotted for the indicated proteins.

## RESULTS

### 53BP1 is required for HR at two-ended DSBs in G2 phase

**Figure 1.**
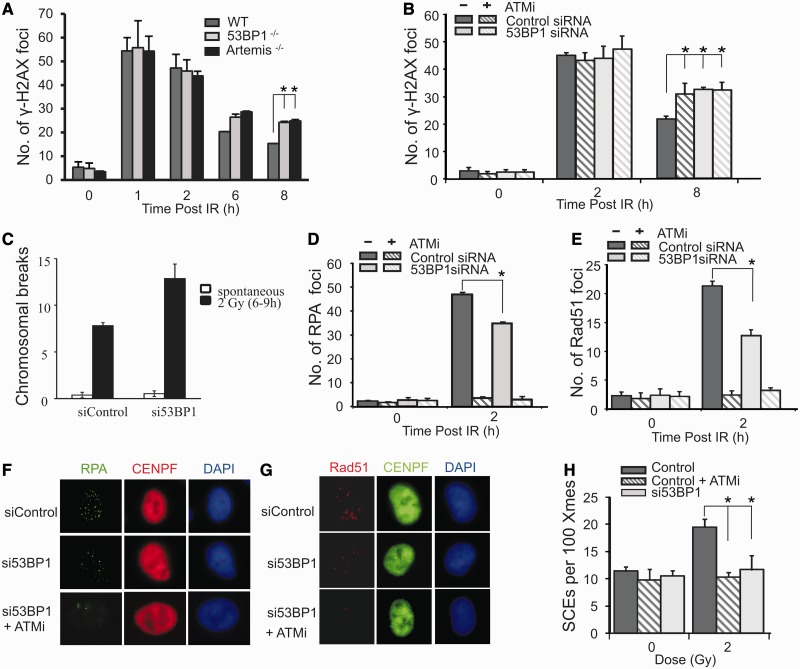
53BP1 promotes DSB repair by HR in G2. (**A**) Wild-type (WT), 53BP1^−/−^ and Artemis^−/−^ MEFs were exposed to 3 Gy IR and γH2AX foci enumerated to 8 h post-IR. G2 cells were identified by CENPF staining (11). Aphidicolin was added before IR to prevent S phase cells progressing into G2 during analysis in all experiments. This also enhanced the distinction between S and G2 cells, as aphidicolin induces pan nuclear γH2AX formation in S phase cells. Control experiments verified that this does not affect HR in G2 (4,11). (**B**) γH2AX foci enumeration in A549 cells with or without siRNA 53BP1 following 3 Gy IR. ATMi was added as indicated. (**C–H**) Chromosomal breaks (C), RPA foci (D), RAD51 foci (E) and SCEs generated from G2 cells (H). RPA and RAD51 foci were enumerated at 2 h post 3 Gy IR. Chromosomal breaks and SCEs were scored at the first metaphase post 2 Gy IR, with or without ATMi and 53BP1 siRNA. To examine SCEs, cells were grown for two cell cycles in BrdU-containing medium. Aphidicolin was added to prevent analysis of S phase cells. Cells were exposed to 2 Gy IR and metaphases collected 12 h post-IR. Caffeine was added at 8 h to relieve the G2/M checkpoint arrest. (F) and (G) show typical images of RPA and RAD51 foci in G2 cells following 53BP1 siRNA with or without ATMi. Control experiments have shown that RPA and RAD51 foci numbers are maximal ∼2 h post-IR (11). Results shown were obtained using a single 53BP1 oligonucleotide; identical results were obtained using a pool of 53BP1 oligonucleotides distinct to the single oligonucleotide (Supplementary Figure S1). Knockdown efficiency is shown in Supplementary Figure S1.

The requirement for 53BP1 for DSB repair in G2 phase was initially examined by enumerating γH2AX foci in irradiated 53BP1^+/+^ and ^−^^/−^ MEFs. In this and all subsequent experiments, cells were treated with aphidicolin, an inhibitor of the replicative polymerases, to prevent progression of S phase cells into G2 and to aid identification of G2 cells. Rigorous controls have shown that this does not affect either NHEJ or HR (4,11). DSB repair was normal at 2 h but impaired by 6–8 h post-IR (Figure 1A), which is similar to the defect observed in 53BP1^−^^/−^ G0/G1 phase MEFs where 53BP1 functions in the ATM signalling pathway leading to HC relaxation (6–8). These findings were verified using siRNA to deplete 53BP1 in A549 cells (Figure 1B). A repair defect was further consolidated using a non-foci method, by monitoring chromosomal breaks in metaphase spreads from cells irradiated in G2 phase (Figure 1C). Increased chromosomal breakage was observed in 53BP1 siRNA cells compared with control cells. Although the repair defect observed is modest and only observed at later times post IR, it is entirely consistent with the findings using G0/G1 phase cells.

The magnitude of the repair defect in 53BP1^−^^/−^ MEFs was similar to that of Artemis^−^^/−^ MEFs, and A549 cells treated with 53BP1 siRNA displayed an epistatic DSB repair defect to cells treated with an ATMi (Figure 1A and B). ATM and Artemis have been shown to function in the slow component of DSB repair which, in G2 phase, represents HR (4). The epistatic relationship observed between 53BP1 and ATM raised the possibility that 53BP1 may also enable DSB repair by HR in G2 phase. To test this possibility, the formation of HR intermediates, namely, RPA and RAD51 foci, was examined in A549 cells at 2 h post-IR with or without siRNA 53BP1. This revealed modestly diminished RPA foci formation and more markedly impaired RAD51 loading following siRNA 53BP1 (Figure 1D–G). ATMi treatment yielded a more marked defect in both end-points (Figure 1D and E). SCEs, a direct consequence of HR, are frequently derived from ATR-dependent HR during replication. We previously established a procedure to examine SCEs in mitotic cells derived from irradiated G2 cells (4). This process involves the analysis of SCEs at 12 h post-IR exposure (in the presence of aphidicolin to prevent progression of S phase cells) and the addition of caffeine to overcome the G2/M checkpoint arrest activated by IR exposure (see ‘Materials and Methods’ section for further details). siRNA 53BP1 abolished SCEs derived from irradiated G2 cells (Figure 1H). Addition of ATMi with or without 53BP1 shows that ATMi similarly abolishes such SCE formation, demonstrating that 53BP1 functions in an ATM-dependent pathway to promote HR (Figure 1H).

**Figure 2.**
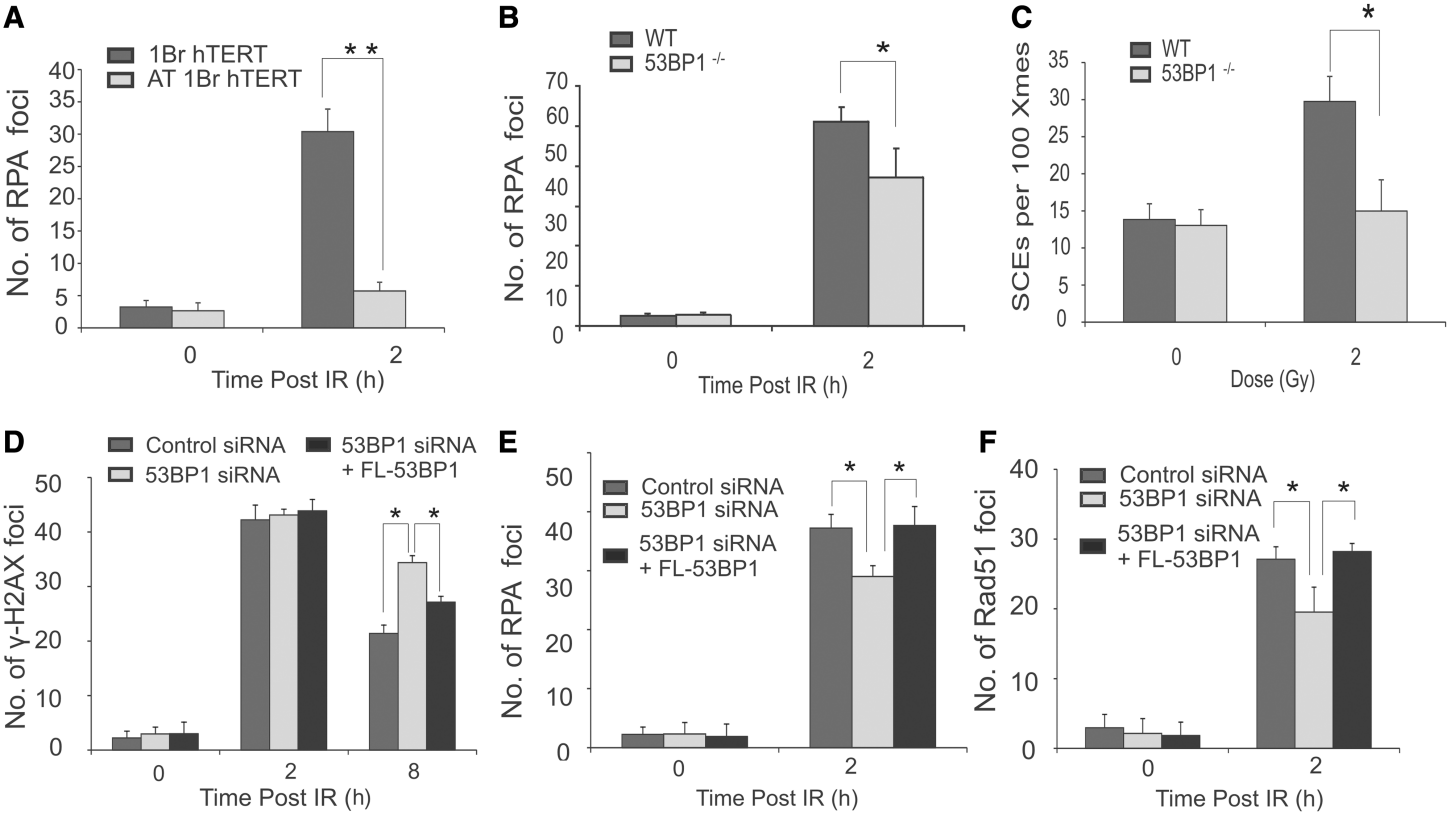
Analysis of HR repair in immortalized human fibroblasts, in mouse embryonic fibroblasts and in complemented tumour cells. (**A**) Immortalized WT and A-T (AT1BR hTERT) deficient human fibroblasts were exposed to 3 Gy IR, and RPA foci were enumerated at 2 h post-IR. G2 cells were identified by CENPF staining. Aphidicolin was added before IR to prevent S phase cells progressing into G2 during analysis in all experiments. (**B–C**) Enumeration of RPA foci (B) and SCEs (C) in Wild-type (WT) and 53BP1^−/−^ MEFs following IR. The statistical significance was determined using Student *t*-test. Asterisks indicate *P* < 0.05, double asterisks indicate *P* < 0.001. (**D–F**) U2OS cells were transfected with an siRNA oligonucleotide for 48 h to deplete 53BP1. Cells were then transfected with a plasmid encompassing an siRNA resistant full-length (FL) 53BP1 cDNA. Twenty-four hours later, cells were irradiated and enumerated for γH2AX (D) RPA (E) or RAD51 (F) foci. Foci were scored in HA^+^ cells, a marker present on the plasmid, providing a marker for cells that had undergone transfection. Results represent the mean and s.d. of three experiments. The statistical significance was determined using Student *t*-test. Asterisks indicate *P* < 0.05.

To substantiate these defects in a manner that does not rely on siRNA-mediated depletion or the addition of ATMi, we firstly exploited AT1BRhTERT cells, an ATM-defective hTERT immortalized cell line, and observed a marked defect in RPA foci formation, demonstrating that the resection defect is due to loss of ATM activity and not a dominant impact of ATMi (Figure 2A). Additionally, we observed diminished RPA foci formation and SCEs in 53BP1^−^^/−^ MEFs (Figure 2B and C).

Finally, to further substantiate these findings, we transfected U20S cells subjected to siRNA 53BP1 with a plasmid expressing siRNA resistant WT 53BP1 cDNA and examined γH2AX, RPA and RAD51 foci in cells expressing the HA plasmid marker (Figure 2D–F). We observed complementation of the 53BP1 defect in each case.

We conclude that although depletion or loss of 53BP1 confers only a modest defect in RPA and RAD51 foci formation, 53BP1 is essential for HR in G2 phase following IR, as SCEs, a direct readout for HR, are diminished, and, in parallel, increased chromosomal breaks are observed.

### HC-DSBs are predominantly repaired by HR in G2 phase

**Figure 3.**
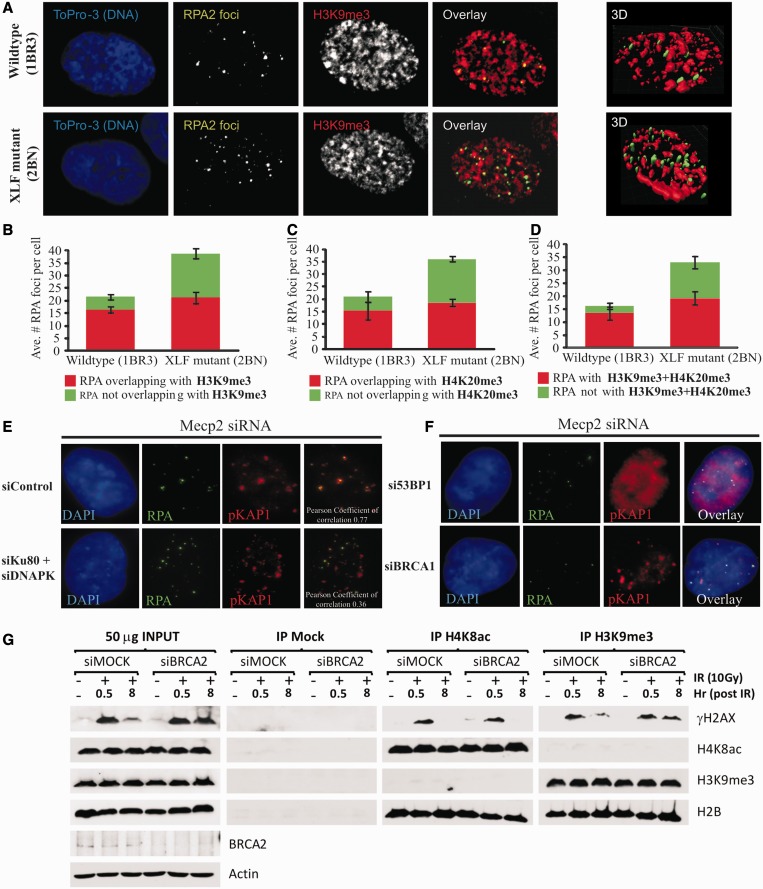
53BP1 is specifically required for HR at HC-DSBs. (A) Visualization of H3K9me3 and RPA foci in G2 cells. Proliferating 1BR3hTERT and 2BNhTERT (XLF deficient) cells were exposed to 3 Gy IR in the presence of aphidicolin. Eight hours post-IR, cells were extracted and immunostained for RPA2 (green), H3K9me3 (red) and DNA (ToPro03, blue). Slides were imaged using a Zeiss confocal microscope by capturing Z-stacks and performing 3D rendering (see Supplementary Figure S3 for details). Far right panels are representative examples of 3D rendered images of 1BR3 (wildtype) and 2BN (XLF mutant) human cells stained for RPA2 (green) and H3K9Me3 (red). (**B–D**) The experiment in (A) was repeated three times with either H3K9me3, H4K20me3 or H3K9me3+H4K20me3 in the red channel. The overlap between RPA foci and HC markers was then quantified by computer analysis (see Supplementary Figure S3), and mean values for three independent experiments for each condition were plotted. (**E**) Co-localization of RPA and pKAP-1 IRIF in G2 phase. 1BR3hTERT cells were harvested 8 h post 3 Gy IR in the presence of aphidocolin and immunostained with the indicated antibodies. Analysis was undertaken in G2 cells; S phase cells were excluded from analysis by their pan-nuclear RPA staining caused by aphidicolin addition. G1 cells do not show RPA foci. G2 cells have low chromatin bound KAP-1 making visualization of pKAP-1 foci analysis difficult (23). To visualize pKAP-1 in G2 cells, cells were subjected to MeCP2 siRNA, which we previously observed causes increased foci expansion and visualization of pKAP-1 in G2 (24). RPA foci show significant overlap with pKAP-1. Similar analysis was carried out after combined Ku80+DNA-PKcs siRNA (DNA-PK siRNA). Under these conditions, the overlap between RPA and pKAP-1 was substantially reduced. (**F**) 53BP1 but not BRCA1 is required for pKAP-1 IRIF in G2 phase F). Analysis was carried out as in Figure 2E following siRNA MeCP2+ 53BP1 or +BRCA1. The 53BP1 is required for pKAP-1 foci formation but is dispensable for pan-nuclear KAP-1 phosphorylation (8). (**G**) Hela cells were subjected to a double thymidine block and released for 7 h. The presence of G2 cells was examined using pS10-H3 antibodies (Supplementary Figure S4). Before release from the double thymidine block, very few pS10-H3^+^ cells were present. At 7 h post-release, most cells are pS10-H3^+^ (Supplementary Figure S4). Controls showing the specificity of these antibodies for HC or EC regions are shown in Supplementary Figure S4B. Hela cells were treated with either scrambled (siMOCK) or BRCA2 (siBRCA2) siRNA and were synchronized to G2 phase by double thymidine blockage. Following 0 or 10 Gy IR, cells were harvested and subjected to IP at 0.5 or 8 h using α-H3K9me3, α-H4K8ac (both from Abcam) or pre-immune IgG (=Mock IP) antibodies. The IP samples were then immunoblotted for the indicated proteins. Persistent γH2AX in BRCA2-depleted cells is predominantly observed in α-H3K9me3 (HC) immunoprecipitates rather than immunoprecipitates obtained using α-H4K8ac (EC).

The finding that 53BP1 promotes HR in G2 phase was surprising given that several studies have shown that it is dispensable for HR and, indeed, that in some situations, loss of 53BP1 can lead to enhanced HR (22). However, we have previously demonstrated that in G0/G1 phase cells, 53BP1 mediates the repair of DSBs located in dense HC regions by promoting ATM-dependent pKAP-1 foci formation and hence HC relaxation (8). We therefore reasoned that 53BP1 might not be an HR factor *per se* but rather enables HC-DSB repair, which in G2 phase occurs by HR. We previously suggested that, after γ- or X-ray exposure, HR in G2 arises predominantly at HC-DSBs, which are repaired with slow kinetics owing to a barrier created by the HC superstructure (4,11). Next, we sought to consolidate the idea that HR occurs predominantly at HC-DSBs. As one approach, we used confocal microscopy and immunofluorescence staining for the HC markers, H3K9me3 and H4K20me3, to visualize HC regions in G2 cells (See Supplementary Figure S2 for details of analysis). At 8 h post-IR in WT cells, >71% of RPA foci overlap with H3K9me3 or H4K20me3 positive chromatin, whereas, when both HC markers were used (to enhance HC demarcation), >88% of RPA foci co-localize with HC (Figure 3A–D). We previously showed that ∼15–20% of DSBs undergo resection in control G2 cells and that the level of resected DSBs increases following loss of NHEJ proteins, suggesting that HR can also occur at EC-DSBs (11). Here, we observed that in 2BN cells, which lack the NHEJ component, XLF, the additional RPA foci persisting 8 h post-IR (compared WITH control) predominantly occur in H3K9me3 or H4K20me3 negative chromatin (i.e. euchromatin) (Figure 3A–D).

Next, we examined the relationship between pKAP-1 foci and HR in G2 phase. In G1 cells, we observed that pKAP-1 foci only form at the subset of DSBs repaired with slow kinetics and that these represent HC-DSBs, where KAP-1 is most abundant (8). Previously, we were unable to detect pKAP-1 foci in G2 cells owing to partial dispersal of KAP-1 from chromocentres in late G2/mitosis (23). We previously reported that Rett Syndrome cells, which display disordered chromatin due to reduced MeCP2 levels, have enlarged γH2AX foci, attributed to reduced chromatin compaction, which allows expansion of IRIF (24). We reasoned that if IRIF were enlarged, then pKAP-1 foci might become visible in G2 phase cells, despite the partial dispersal of KAP-1 from chromocentres. Indeed, following siRNA MeCP2, pKAP-1 foci were visible in G2 cells (Figure 3E). In G0/G1 cells, we have observed that pKAP-1 foci only form at HC-regions (8). Using siRNA MeCP2, we found that RPA foci substantially co-localize with pKAP-1 foci in G2 cells (Figure 3E). Additionally, following combined Ku80+DNA-PKcs siRNA (siRNA DNA-PK), this overlap is greatly diminished. As, shown previously, RPA foci co-localize with the HC markers, H3K9me3/H4K20me3, this supports the notion that pKAP-1 foci predominantly form at HC-DSBs in G2 (as in G1). This analysis strongly argues that HR occurs primarily at HC-DSBs in WT cells but can arise at additional DSBs, which we suggest represent EC-DSBs, when DNA-PK is absent.

In G1 phase cells, pKAP-1 foci formation is ATM and 53BP1 dependent. Since pKAP-1 foci could be observed in G2 cells following MeCP2 siRNA, we examined whether such foci were also dependent on 53BP1 in G2 phase. Strikingly, pKAP-1 foci did not form in G2 (as in G1) cells following 53BP1+MeCP2 siRNA (Figure 3F).

We also used an immunoprecipitation approach to consolidate the notion that HR predominantly repairs HC-DSBs. In this method, the distribution of γH2AX, which forms at DSB sites, was examined in histones derived from HC or EC regions following IP using H3K9me3 or H4K8Ac antibodies, respectively. A G2-phase enriched population of cells was exposed to 10 Gy IR. At 30 min post-IR, but not in the 0 Gy control, γH2AX was enriched on histones pulled down with either H3K9me3 or H4K8ac, as expected for the random distribution of IR induced DSBs between HC and EC. By 8 h, γH2AX was no longer detectable in the histones obtained using α-H4K8Ac antibodies (i.e. EC-enriched histones), suggesting that EC-DSBs have largely undergone repair, whereas residual γH2AX was detectable in the α-H3K9me3 immunoprecipitates consistent with the notion that HC-DSBs are repaired slowly (Figure 3G). To examine the DSB repair process, this analysis was carried out following siRNA BRCA2, an HR protein. Importantly, although γH2AX in the α-H4K8Ac immunoprecipitates disappeared independently of BRCA2 depletion, γH2AX persisted in the α-H3K9me3 immunoprecipitates, consistent with the notion that HC-DSBs require BRCA2-dependent HR for repair (Figure 3G).

**Figure 4.**
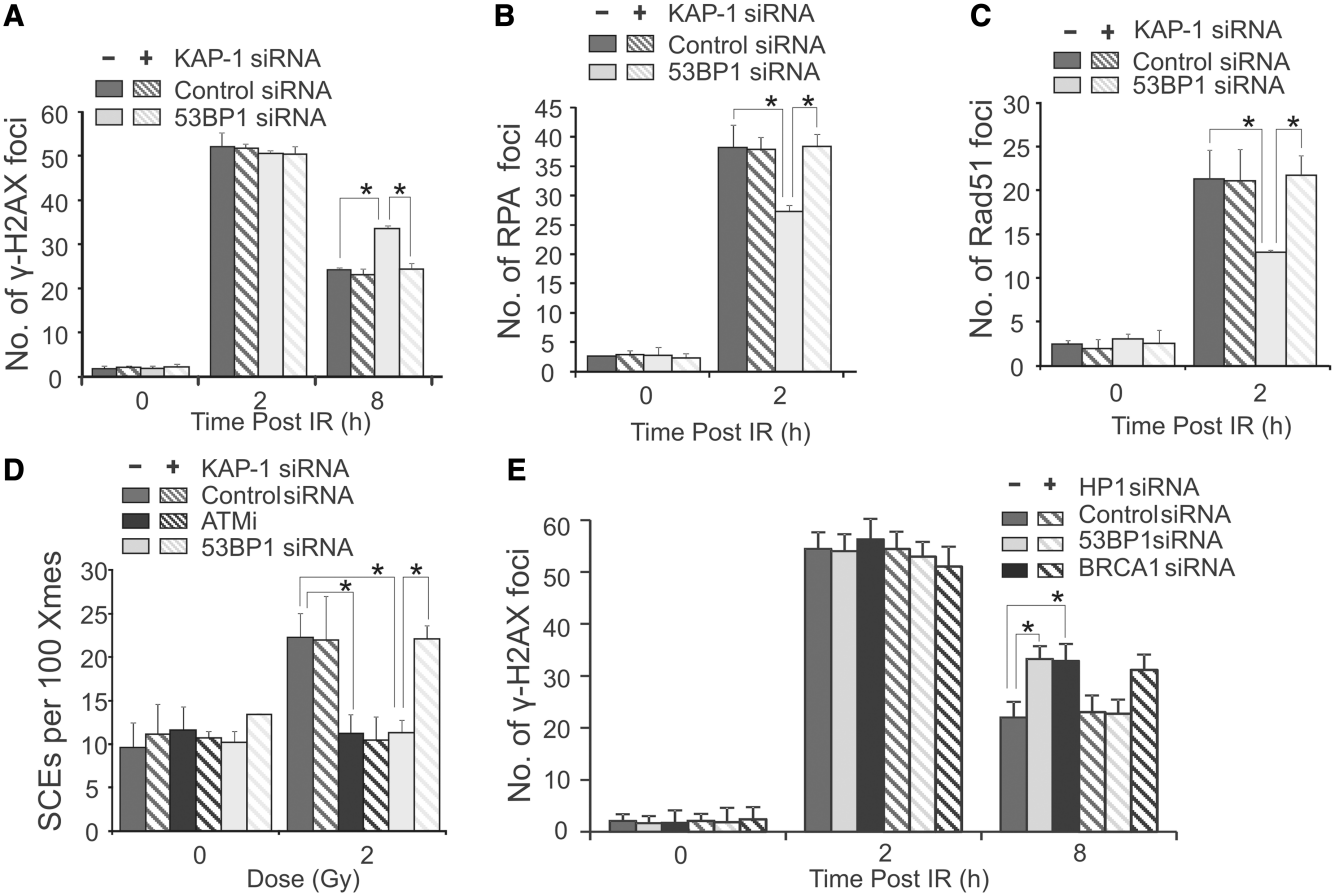
The defect in HR in 53BP1 siRNA cells can be relieved by KAP-1 or HP1 siRNA. γH2AX (**A**), RPA (**B**) or RAD51 (**C**) foci were enumerated in A549 as indicated following 3 Gy IR with or without siRNA KAP-1. (**D**) Quantification of SCEs in mitotic cells arising from irradiated G2 cells. A pool of 53BP1 oligonucleotides was used in this and all following experiments as efficient knockdown was achieved (Supplementary Figure S1A). The efficiency of siRNA KAP-1 is shown in Supplementary Figure S1B. (**E**) A549 cells were treated with control, 53BP1 or BRCA1 siRNA with or without siRNA to all three isoforms of HP1 (α,β,γ) and irradiated with 3Gy IR. Analysis was carried out as in (A). For all endpoints, siRNA KAP-1 or HP1 relieved the defects observed by siRNA 53BP1. Results represent the mean and s.d. of three experiments. The statistical significance was determined using Student’s *t*-test. Asterisks indicate *P* < 0.05.

To further consolidate the notion that 53BP1 is required for HR by promoting HC relaxation, we examined whether HC relaxation caused by knockdown of HC factors can overcome the requirement for 53BP1 for DSB repair in G2. Strikingly, siRNA KAP-1 restored normal γH2AX foci loss, resection (assessed by RPA foci numbers), RAD51 loading and SCE formation to 53BP1-depleted G2 cells (Figure 4A–D). Knockdown of HP1 (α,β + γ) similarly overcame the need for 53BP1 for G2 phase DSB repair (Figure 4E). Collectively, these findings substantiate the notion that HR occurs predominantly at HC regions in control G2 phase cells and that relaxing HC by knockdown of HC factors overcomes the need for 53BP1 for DSB repair in G2 phase. This suggests that 53BP1’s role in HR in G2 phase lies in promoting the requisite HC relaxation.

### 53BP1 is dispensable for HR following depletion of DNA-PK or following treatment with MMS or HU

**Figure 5.**
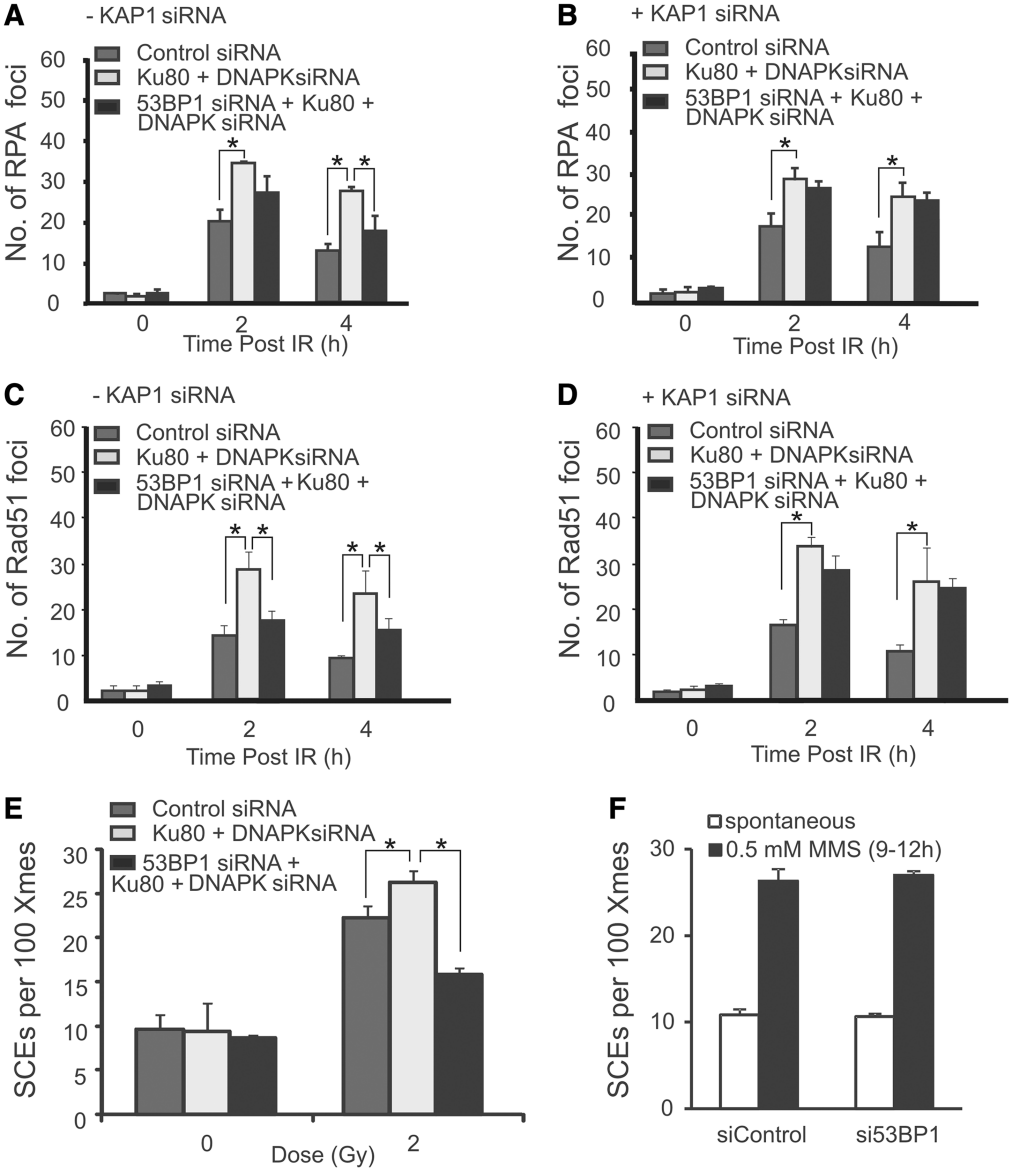
53BP1 is dispensable for HR following siRNA DNA-PK, and for HR in S phase. (**A, B**) RPA foci were enumerated in G2 cells at the indicated times after exposure to 1 Gy IR in A549 cells subjected to control siRNA, siRNA Ku80+DNA-PKcs or siRNA 53BP1+Ku80+DNA-PKcs either without (A) or with (B) KAP-1 siRNA. (**C, D**) As shown for panels (A–B) but RAD51 foci are shown instead of RPA foci. Results following 53BP1 siRNA alone are given in Figure 2. Results represent the mean and s.d. of three experiments. (**E**) SCEs in G2 phase A549 cells following exposure to 2 Gy IR. Results represent the mean and s.d of three experiments. (**F**) SCEs in A549 cells treated with 0.5 mM MMS for 0.5 h. No reduction is SCEs resulting from MMS treatment was observed following 53BP1 siRNA. Results represent the mean and s.d. of three experiments. The statistical significance was determined using Student’s *t*-test. Asterisks indicate *P* < 0.05. Confirmation of 53BP1 knockdown efficiency following siRNA DNA-PK is shown in Supplementary Figure S1.

Although DSBs in EC regions (EC-DSBs) are repaired predominantly by NHEJ, they can be forced to undergo repair by HR following depletion of NHEJ proteins. Following Ku80+DNA-PKcs siRNA, DSB repair is slowed, and increased RPA and RAD51 foci numbers are observed (Figure 5A and C) and increased G2 phase SCEs arise (Figure 5E) i.e. DNA-PK depletion results in enhanced HR. Using this method, we examined whether 53BP1 was required for HR following DNA-PK depletion to establish whether 53BP1 is a core HR component and therefore required for all HR events. siRNA Ku80+DNA-PKcs+ 53BP1 compared with siRNA Ku80+DNA-PKcs (i.e. without 53BP1 siRNA) revealed a modest reduction in RPA foci (Figure 5A and B), RAD51 foci (Figure 5C and D) and SCEs (Figure 5E). This reduction was similar to that observed in cells treated only with siRNA 53BP1 (see Figure 1). As in Figure 1, the magnitude of the reduction was greater for RAD51 than for RPA foci. Further, following additional depletion of KAP-1 (i.e. DNA-PK/53BP1/KAP-1 siRNA), RPA foci numbers were identical to that observed in cells subjected to DNA-PK siRNA alone (Figure 5B). Similar results were obtained monitoring RAD51 foci (Figure 5C and D). (NB SCE analysis was not carried out following quadruple knockdown due to a diminished mitotic index.) Collectively, these results suggest that 53BP1 is dispensable for the additional level of HR that arises following DNA-PK depletion but promotes HR at HC-DSBs via its role in promoting HC relaxation. Similarly, depletion of XLF, another NHEJ protein, resulted in an enhanced level of RPA and RAD51 foci, consistent with an enhanced level of repair by HR, and this was also observed following co-depletion of XLF, 53BP1 and KAP-1 (Supplementary Figure S4A and B). These data show that 53BP1 is not an essential HR component, as HR can occur following depletion of 53BP1 at the persistent DSBs that accrue in the absence of NHEJ proteins.

To consolidate these findings, we examined the requirement of 53BP1 for SCE formation in cells treated with DNA-damaging agents in S phase. SCEs have been shown to arise following replication in the presence of the alkylating agent, MMS (25). As MMS does not induce DSBs in G1 phase cells, it is likely that SCEs are generated by HR following MMS-induced replication fork stalling or collapse. We predicted that such HR might not require HC relaxation if it arises during replication when the HC superstructure is transiently dismantled. Consistent with this, 53BP1 was dispensable for SCE formation following MMS treatment (Figure 5F). Further consolidating the notion that 53BP1 is dispensable for HR arising during replication, we show that 53BP1 depletion causes enhanced (and not diminished) HR following HU treatment (Supplementary Figure S4C).

Collectively, these findings substantiate and extend previous data, suggesting that after IR, HR occurs predominantly at HC regions. We propose that HC represents a partial barrier to resection and a full barrier to the completion of HR (SCEs) at DSBs in G2 and that 53BP1 promotes pKAP-1 foci formation to relieve that barrier. As HR occurs predominantly at HC regions after IR, 53BP1, which is required for HC relaxation, promotes this form of HR. However, HR can occur efficiently without 53BP1 at HC-DSBs following siRNA of HC proteins (KAP-1 or HP1), at the additional DSBs that undergo HR following DNA-PK depletion, and in S phase following MMS or HU treatment. Moreover, 53BP1 is dispensable for NHEJ because the fast DSB repair process (NHEJ) takes place in 53BP1^−^^/−^ cells (Figure 1A). This strongly suggests that 53BP1 is dispensable for both HR and NHEJ but can promote either process by promoting HC relaxation at HC-DSBs.

### Co-depletion of 53BP1 and KAP-1 restores HR in BRCA1 knockdown cells

Having examined 53BP1’s role in DSB repair, we next examined the requirement for BRCA1 and its interplay with 53BP1 during G2 phase HR. First, we monitored whether BRCA1, like 53BP1 and ATM, also impacts on DSB repair in HC regions via pKAP-1-mediated chromatin relaxation. Importantly, we had observed normal pKAP-1 foci formation in G2 phase cells following siRNA BRCA1+MeCP2 in contrast to the defect observed following siRNA 53BP1 (Figure 3F), suggesting that BRCA1 is dispensable for pKAP-1 foci formation and hence HC relaxation.

**Figure 6.**
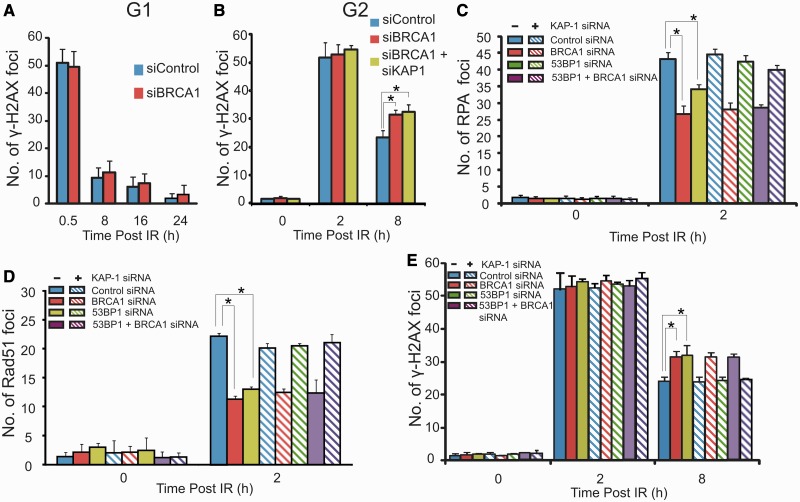
BRCA1 is dispensable for HC-DSB repair while combined loss of BRCA1, 53BP1 and KAP-1 allows DSB repair by HR. (**A, B**) A549 cells were exposed to 3 Gy IR and γH2AX foci enumerated in G1 and G2 cells with or without siRNA BRCA1 and KAP-1. G2 and G1 cells were identified as in Figure 1. Results shown used a single BRCA1 oligonucleotide; identical results were obtained using a distinct pool of oligonucleotides**.** Aphidicolin was added as previously described. (**C–E**) A549 cells were treated with the indicated siRNAs, exposed to 3 Gy IR and examined for RPA (C), RAD51 (D) or γH2AX (E) foci at the indicated times. Results are shown in samples with or without siRNA KAP-1 to expose the necessity to have relaxed HC to observe HR in G2 phase. The k.d efficiency following multiple siRNA transfection is shown in Supplementary Figure S1. Results represent the mean and s.d. of three experiments. The statistical significance was determined using Student’s *t*-test. Asterisks indicate *P* < 0.05.

To gain further insight, we examined the impact of BRCA1 depletion on DSB repair. In G1 cells, we observed normal γH2AX foci loss post IR (fast and slow components) following siRNA BRCA1 but reproducibly observed defective DSB repair in BRCA1-depleted G2 cells detectable as a failure to undergo the slow DSB repair process (Figure 6A and B). The fact that the slow DSB repair process in G1 (HC-DSB repair) is BRCA1 independent is consistent with the finding above that BRCA1 is dispensable for pKAP-1 foci formation. The failure to repair the slow component of DSB repair in G2 (which, we argue, represents HR) is consistent with the substantial evidence that BRCA1 is essential for HR (26–29). Consistent with the notion that BRCA1’s role in DSB repair in G2 represents a direct function in HR rather than HC relaxation, we found that combined siRNA BRCA1+KAP-1 did not overcome the repair defect caused by siRNA BRCA1 in contrast to the findings with 53BP1 (Figure 6B).

Recent findings have shown that combined depletion of 53BP1 and BRCA1 enables resection and HR to proceed following replication stalling (12,13). We, therefore, examined whether combined depletion of 53BP1 and BRCA1 enables resection and HR to proceed at IR-induced DSBs in G2 phase, bearing in mind that this represents HR at HC-DSBs (12,13). In contrast to the situation in S phase cells, combined knockdown of 53BP1 and BRCA1 does not restore RPA or RAD51 foci formation or DSB repair (Figure 6C–E). However, consistent with 53BP1’s role in promoting HC relaxation, triple knockdown of 53BP1, BRCA1 and KAP-1 restored all these end points to control levels (NB SCE formation was not examined following triple knockdown due to a diminished mitotic index) (Figure 6C–E). These findings suggest that 53BP1 has two functions in G2, a positive role in promoting HC relaxation and an inhibitory function that is overcome by BRCA1. The first function is consistent with 53BP1’s role in mediating HC relaxation, whereas the second function is consistent with published findings demonstrating that 53BP1 poses a barrier to resection and HR. Although BRCA1’s function is dispensable for HC relaxation and therefore for DSB repair by NHEJ in G1phase, it is required for HC DSB repair in G2 phase where it overcomes the inhibitory function of 53BP1 and allows HR to proceed.

## DISCUSSION

Previous studies have shown that 53BP1 functions as a pro-NHEJ factor and is inhibitory to HR (12,13). These previous studies have examined HR at one-ended DSBs generated during S phase or have exploited reporter constructs (integrated or as plasmid DNA). In these situations, HR likely occurs in euchromatin regions. Consistent with these previous studies, our findings here show that 53BP1 is dispensable for HR at euchromatin regions. However, contrary to the suggestion that 53BP1 is dispensable for HR, we find that 53BP1 is necessary to promote HR specifically at HC-DSBs in G2 phase cells. Our findings, therefore, do not refute the previous work but rather add an additional role for 53BP1 that is not observed when HR in S phase cells is examined.

Previously, we have shown that in G1 phase, DSBs are repaired with two component kinetics, that the slow component represents the repair of HC-DSBs and that 53BP1 promotes HC-DSB repair by enabling ATM-dependent pKAP-1 foci formation (8). DSBs are also repaired with fast and slow kinetics in G2 phase. However, in G2 the slow component represents HR with evidence, suggesting that the DSBs repaired by HR are predominantly HC-DSBs (11). First, we substantiate that notion. Using H3K9me3 and H4K20me3 to identify chromodomains in G2, we show that RPA foci form predominantly at such domains. Additionally, by exploiting the ability to visualize pKAP-1 foci in G2 following siMeCP2, which allows chromatin relaxation, we show that pKAP-1 foci are 53BP1 dependent and predominantly co-localize with RPA foci. However, depletion of NHEJ components allows RPA foci to form at non-HC regions; under such conditions, RPA and pKAP-1 foci do not co-localize. Finally, using IP and siRNA BRCA2, we show that depletion of BRCA2 dramatically impairs the loss of the DSB marker, γH2AX, at 8 h post-IR in immunoprecipitates enriched for HC histones in contrast to those enriched for EC histones.

Surprisingly, in contrast to previous suggestions that 53BP1 promotes NHEJ, we show that it is required for HR at HC-DSBs in G2. However, and consistent with previous studies, 53BP1 is dispensable for HR in S phase, as well as in G2 phase following depletion of DNA-PK. Further, the role of 53BP1 in HR correlates with its requirement for pKAP-1 foci formation. Thus, although not a core HR protein, we suggest that 53BP1 can indirectly promote HR by enabling HC-relaxation. Consistent with the notion that 53BP1 is specifically required to promote HC relaxation, we find that HR can proceed without 53BP1 following depletion of HC proteins (KAP-1 or HP1). This role of 53BP1 is dispensable during replication, where HC may dismantle, as shown by normal or enhanced SCE formation following MMS or HU treatment, respectively. This is consistent with a previous study showing enhanced SCE formation after HU treatment in cells depleted for 53BP1 (22). Studies on whether 53BP1 inhibits HR in control cells where BRCA1 functions are somewhat conflicting. Clearly, in the absence of BRCA1, HR does not proceed owing to the inhibitory role of 53BP1. In our studies in G2 phase cells, we find that siRNA of 53BP1 leads to diminished RPA and RAD51 foci and SCE formation, which can be relieved by siRNA KAP-1. However, depletion of KAP-1 and 53BP1 does not lead to enhanced SCE formation, suggesting that any inhibitory role of 53BP1 is entirely relieved by KAP-1.

BRCA1 loss causes a specific DSB repair defect in G2, but, unlike 53BP1, BRCA1 is dispensable for pKAP-1 foci formation. A recent study reported that BRCA1 loss impacts on heterochromatinization (30). We previously observed normal DSB repair rates in other human syndromes with disordered HC (e.g. Rett Syndrome), but such cells display a diminished requirement for ATM for HC-DSB repair (7,24). The specific requirement for BRCA1 in G2 is, therefore, a distinct phenotype. Further, we have observed that BRCA1-depleted cells display the anticipated DSB repair defect following ATMi addition in G1 and G2. Thus, any change in HC structure caused by BRCA1 depletion does not impact on the need for ATM for HC-DSB repair.

**Figure 7.**
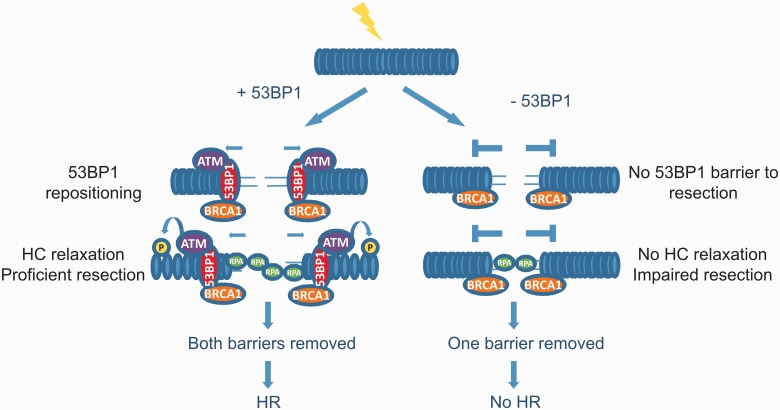
Model showing 53BP1’s opposing roles in G2 phase HR. (**A**) Following DSB induction in G2 phase, 53BP1 localizes to DSB ends and inhibits DNA end resection. At DSBs located at HC regions, BRCA1 overcomes the inhibitory barrier of 53BP1 to resection by excluding 53BP1from these regions. Concomitantly, 53BP1 is retained on chromatin and overcomes the barrier that HC poses to resection by tethering activated ATM and thus mediating HC relaxation via robust KAP-1 phosphorylation. Once the barriers posed to resection by 53BP1 and HC are overcome, DSB repair proceeds by HR. (**B**) In the absence of 53BP1, BRCA1’s function in overcoming 53BP1’s barrier to resection is redundant. However, when 53BP1 is absent, ATM cannot be tethered at the DSB sites, and hence the barrier posed to resection by HC cannot be overcome. Under these conditions, some resection can occur, but it is inefficient due to the HC barrier. Consequently, resection stalls and impaired DSB repair by HR is observed.

To summarize, previous studies have shown that 53BP1 has an inhibitory impact on resection and RAD51 loading, which is relieved by BRCA1 (12,13). Thus, HR stalls in the absence of BRCA1 and, in S phase cells, can progress when both BRCA1 and 53BP1 are absent (Figure 7). In contrast, in G2 phase cells, there is also a pro-HR role for 53BP1, which represents its contribution to the relaxation of HC. Nonetheless, 53BP1 still retains its inhibitory impact on HR, and thus a role for BRCA1 is retained in S and G2 cells. Interestingly, a recent study (and our own unpublished observations) has provided evidence that 53BP1 becomes re-localized to the periphery of enlarged IRIF as HR pursues in G2 phase cells (20). This repositioning is BRCA1 dependent. Thus, we suggest that in G2 phase cells, 53BP1 has both an inhibitory and a promoting role for HR (Figure 6 and Supplementary Figure S2). The inhibitory role is relieved by BRCA1-dependent repositioning of 53BP1 to the periphery of enlarged γH2AX foci. Indeed, we propose that 53BP1 is not simply degraded at IRIF but undergoes defined repositioning to the periphery of IRIF to facilitate the formation of pKAP-1 foci formation to allow HC relaxation at HC-DSBs, which is a prerequisite for HR at such DSBs. This role, however, is not required at EC-DSBs or in S phase cells. These findings are, therefore, consistent with the described role for 53BP1 but provide evidence for an additional role that has not hitherto been described.

## SUPPLEMENTARY DATA

Supplementary Data are available at NAR Online.

## FUNDING

MRC programme grant [G1000050 and G0500897]; the Association for International Cancer Research; the Welcome Research Trust and the EMF Biological Research Trust (to the P.A.J. laboratory); Deutsche Forschungsgemeinschaft [Lo 677/4-3 and GRK1657]; and the Bundesministerium für Bildung und Forschung [03NUK001C, 02NUK016D] (to the M.L. laboratory); Spanish Ministry of Science and Innovation [SAF2010-22357 and CONSOLIDER-Ingenio 2010 CDS2007-0015 to R.F.]; Canadian Institutes of Health Research and the Alberta Cancer Foundation (to A.A.G.). Funding for open access charge: University of Sussex.

*Conflict of interest statement*. None declared.

## Supplementary Material

Supplementary Data
