# Supplementary material for: Opposing roles for 53BP1 during homologous recombination
Source: Nucleic Acids Res. 2013 Aug 22;41(21):9719–31. doi: 10.1093/nar/gkt729 (PMC3834810; doi:10.1093/nar/gkt729)
Supplement: Supplementary Data [file supp_41_21_9719__index.html]

Opposing roles for 53BP1 during homologous recombination — Opposing roles for 53BP1 during homologous recombination — Supplementary Data 

# Opposing roles for 53BP1 during homologous recombination

## Supplementary Data

files

**Files in this Data Supplement:**

- Supplementary Data - pdf file
