# Supplementary material for: Opposing roles for 53BP1 during homologous recombination
Source: Nucleic Acids Res. 2013 Aug 22;41(21):9719–31. doi: 10.1093/nar/gkt729 (PMC3834810; doi:10.1093/nar/gkt729)
Supplement: Supplementary Data [file supp_gkt729_nar-01497-d-2013-File009.pdf]

Supplementary Figure 1: Controls for siRNA knockdown efficiencies.

4 x 10^5 logarithmically growing cells were used for each knockdown and were transfected with siRNA duplexes at a final concentration of 20 nM. Cells were then grown for 72 h prior to fixation and immunostaining/immunoblotting with the indicated antibodies. A) Knockdown efficiency in A549 cells following treatment with either a single or a pool of 53BP1 siRNA oligonucleotides (the single oligonucleotide was distinct to any in the pool). B) As above but knockdown efficiency of KAP-1 siRNA was assessed under single, double and triple knockdown conditions. C-D) BRCA1 single and pool oligonucleotide knockdown efficiency was assessed by immunoblotting and immunostaining. For immunoblotting, MCM6 was used as a loading control. E) 53BP1 knockdown efficiency was monitored by IF following, single, double, triple and quadruple knockdown conditions. 53BP1 levels were greatly diminished in all cases although in the triple and quadruple knockdown conditions slightly more 53BP1 remained. F-I) 53BP1, KAP-1, DNAPK and KAP-1 knockdown efficiencies were assessed by western blotting, following single and multiple knockdown conditions. Ku80, α-tubulin and KAP-1 were used as loading controls.

Supplementary Figure 2.

(A) Highly resolved images of multiple focal planes through the nucleus (z-stacks) were taken using a confocal Zeiss LSM 510 microscope. Z-stacks were further processed using 3D rendering. (B) 3D model of 3D rendered z-stacks with rotation in z-axis. Threshold setting is required in order to analyze foci number. (C) 3D rendering converts the 3D model into a 2D image. The resulting 2D images were analyzed using Image J. The Analysis involves setting the threshold in each image for the heterochromatin and the RPA-foci and capturing the total number of RPA2 foci and the overlap of RPA2 foci and heterochromatin (setting a minimal size for the foci as a requirement).

Supplementary Figure 3. Controls for IP experiments.

A) HeLa cells were synchronized using a double thymidine block. 2 mM thymidine was added to adherent HeLa cells. 15h later, the cells were washed and incubated in thymidine free media for 8h. 2 mM thymidine was then re-added, and the cells were grown for a further 15h. At this point, the cells were highly synchronized in G1 phase (left panel) (no $pH3^+$ cells). Upon removal of thymidine and the addition of fresh media, the cells progressed through the cell cycle in synchrony and approximately 70% of cells were in G2 phase at 7 h post thymidine release, as assessed by H3S10p staining (right panel).

B) Verification that α-H4K9Ac and α-H3K9Me3 detect euchromatin versus heterochromatin,, respectively. NIH3T3 cells were stained with either ChIP-grade α-H4K9Ac (red) or α-H3K9Me3 (green) antibodies and DAPI (blue). Staining of each marker is mostly mutually exclusive and α-H3K9me3 staining occurs at DAPI chromocentres.

Supplementary Figure 4. 53BP1 is dispensable for HR following siRNA XLF and inhibits HU induced SCEs.

A-B) RPA and Rad51 foci were enumerated in G2 cells (treated with the indicated siRNA) 2 h after exposure to 1.5 Gy IR. Results represent the mean and s.d. of three experiments.

C) SCEs in Wild type (WT) and $53BP1^{-/-}$ MEFs following HU. In these experiments using MEFs, we observed enhanced SCEs in the untreated control compared to the untreated control in Figure 2C. We attribute this difference to the addition of aphidicolin to cells in Figure 2C but not here (Fig. S4C). With MEFs, we have observed that aphidicolin does not fully block the progression of S phase cells, with a small proportion of cells progressing to G2/mitosis with damage. For SCE analysis, where cells are harvested 12 h post IR, a small proportion of S phase cells appear to reach mitosis, enhancing the background SCE level. The progression of S phase cells was not observed in aphidicolin treated A549 cells. However,

this does not influence the marked increase in IR-induced SCEs, which arise in the predominantly G2 phase cells and the reduction in 53BP1 deficient MEFs .

D) Full list of target sequences from the Dharmcacon ON-TARGETplus SMARTpool,

Supplementary Figure 5: **Model showing 53BP1's role(s) in G2 phase HR and in S-phase HR mediated fork restoration.**

A-B) DSB repair by HR in G2 phase. A) Following DSB induction in G2 phase, 53BP1 localises to DSB ends and inhibits DNA end resection. At DSBs located at HC regions, BRCA1 overcomes the inhibitory barrier of 53BP1 to resection by excluding 53BP1from these regions. Concomitantly, 53BP1 is retained on chromatin and overcomes the barrier that HC poses to resection by tethering activated ATM and thus mediating HC relaxation via robust KAP-1 phosphorylation. Once the barriers posed to resection by 53BP1 and HC are overcome, DSB repair proceeds by HR. B) In the absence of 53BP1, BRCA1's function in overcoming 53BP1's barrier to resection is redundant. However, when 53BP1 is absent ATM cannot be tethered at the DSB sites and hence the barrier posed to resection by HC cannot be overcome. Consequently, resection stalls and impaired DSB repair by HR is observed.

C-D) DSB repair by HR in S-phase. C) Following replication fork stalling/collapse in S-phase, 53BP1 localises to these regions and inhibits DNA end resection. BRCA1 functions to overcome the inhibitory barrier of 53BP1 to resection thus allowing fork restoration by HR to proceed. D) In the absence of 53BP1, BRCA1's function in overcoming 53BP1's barrier to resection is redundant. Moreover, 53BP1's role in chromatin relaxation is also redundant since during replication the HC superstructure is transiently dismantled and does therefore

not pose a barrier to resection. Therefore unlike the situation in G2 phase where HR stalls in the absence of 53BP1, in S-phase, resection and HR can take place in the absence of 53BP1.

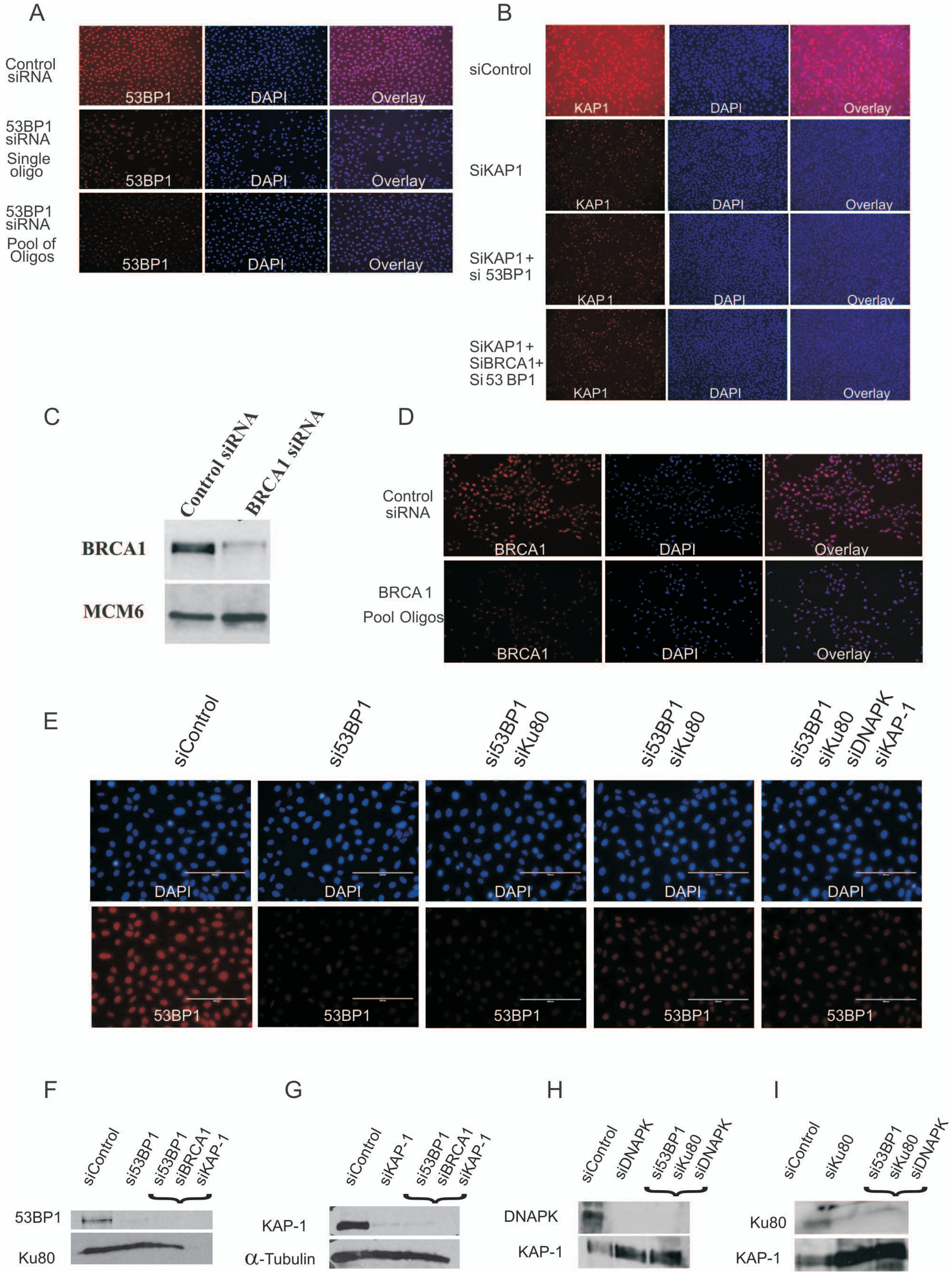
A
Control siRNA
53BP1 siRNA Single oligo
53BP1 siRNA Pool of Oligos
53BP1
DAPI
Overlay
B
siControl
SiKAP1
SiKAP1+ si 53BP1
SiKAP1+ SiBRCA1+ Si 53 BP1
KAP1
DAPI
Overlay
C
Control siRNA
BRCA1 siRNA
BRCA1
MCM6
D
Control siRNA
BRCA 1 Pool Oligos
BRCA1
DAPI
Overlay
E
siControl
si53BP1
si53BP1 siKu80
si53BP1 siKu80 siDNAPK siKAP-1
DAPI
53BP1
F
siControl
si53BP1
si53BP1 siBRCA1 siKAP-1
53BP1
Ku80
G
siControl
siKAP-1
si53BP1 siBRCA1 siKAP-1
KAP-1
α-Tubulin
H
siControl
siDNAPK
si53BP1 siKu80 siDNAPK
DNAPK
KAP-1
I
siControl
siKu80
si53BP1 siKu80 siDNAPK
Ku80
KAP-1

Figure S1

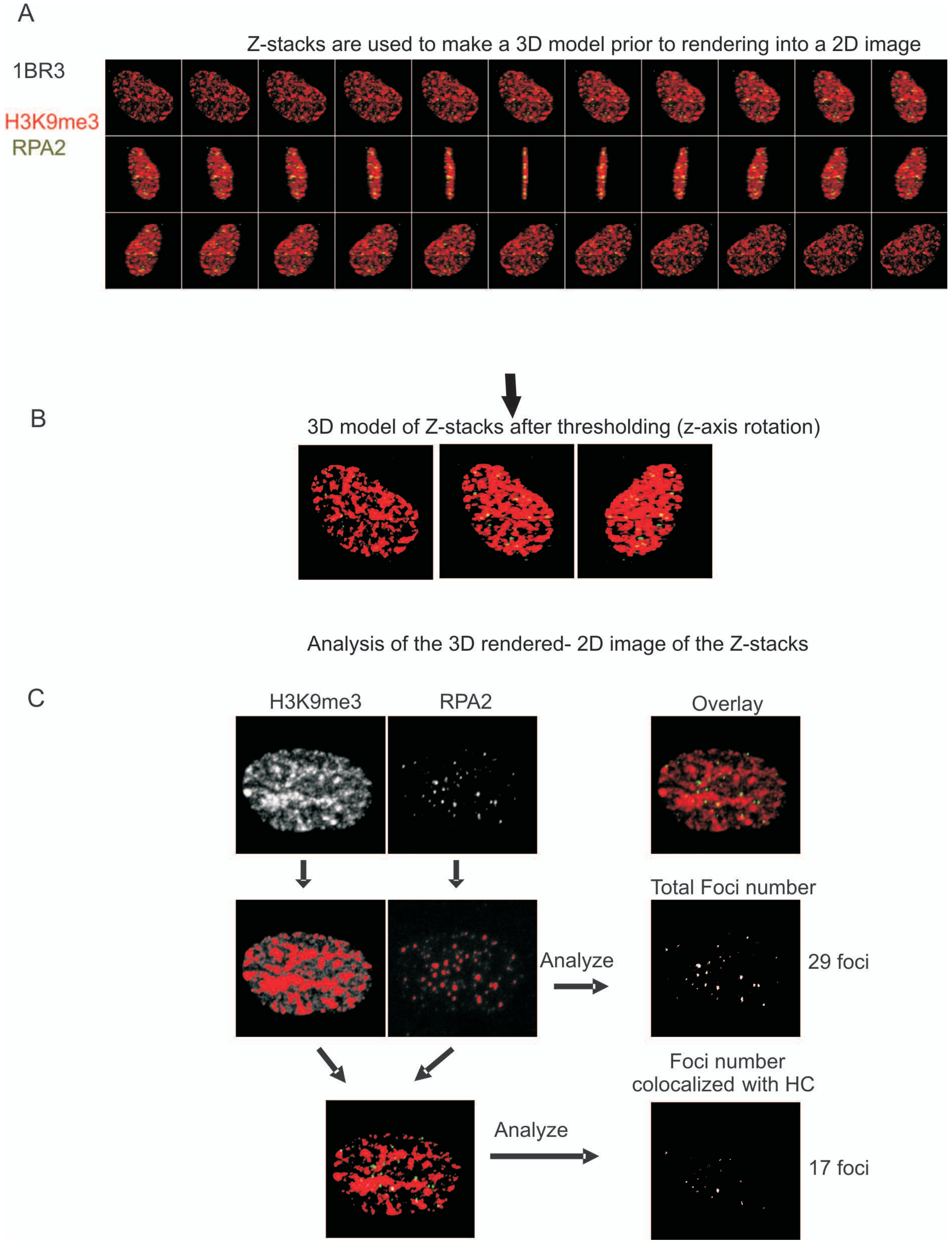
A
Z-stacks are used to make a 3D model prior to rendering into a 2D image
1BR3
H3K9me3
RPA2
B
3D model of Z-stacks after thresholding (z-axis rotation)
Analysis of the 3D rendered- 2D image of the Z-stacks
C
H3K9me3
RPA2
Overlay
Total Foci number
Analyze
29 foci
Foci number
colocalized with HC
Analyze
17 foci

Figure S2

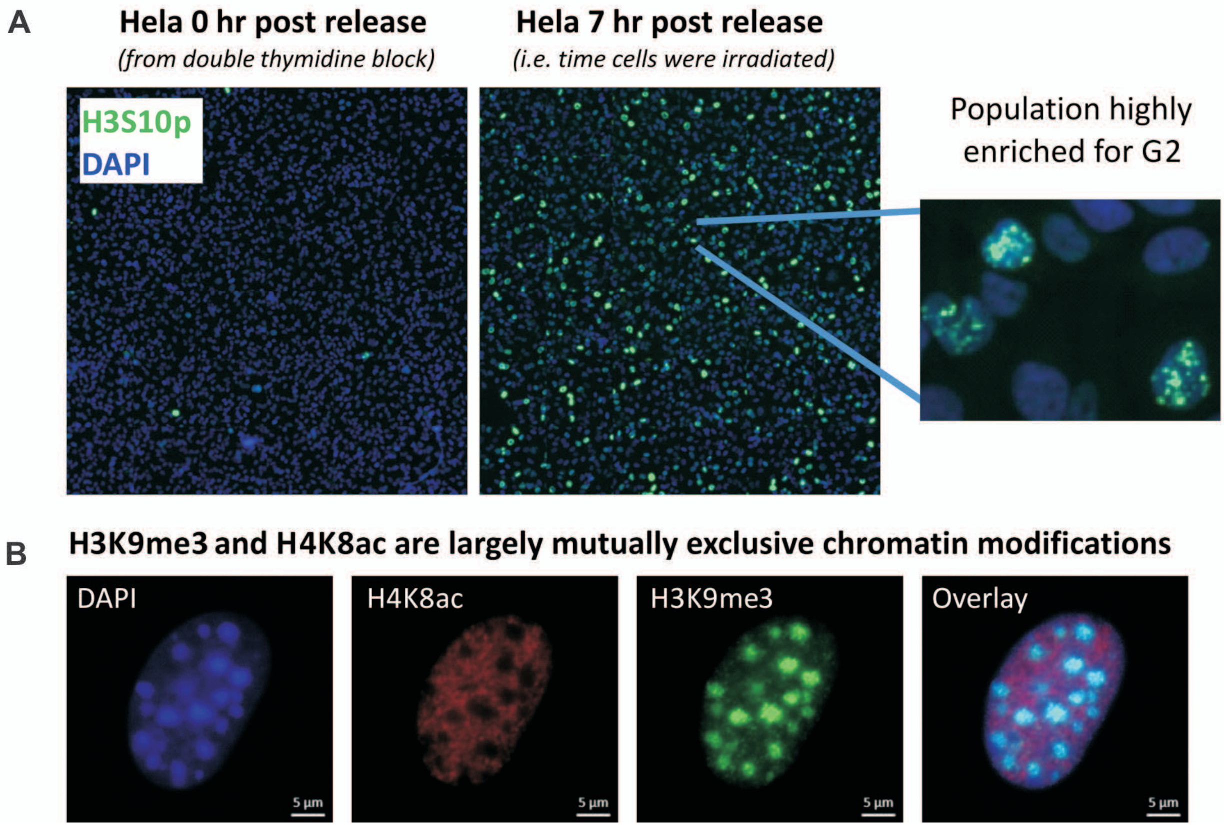
A
Hela 0 hr post release
(from double thymidine block)
Hela 7 hr post release
(i.e. time cells were irradiated)
H3S10p
DAPI
Population highly enriched for G2
B
H3K9me3 and H4K8ac are largely mutually exclusive chromatin modifications
DAPI
H4K8ac
H3K9me3
Overlay
5 µm
5 µm
5 µm
5 µm

Figure S3

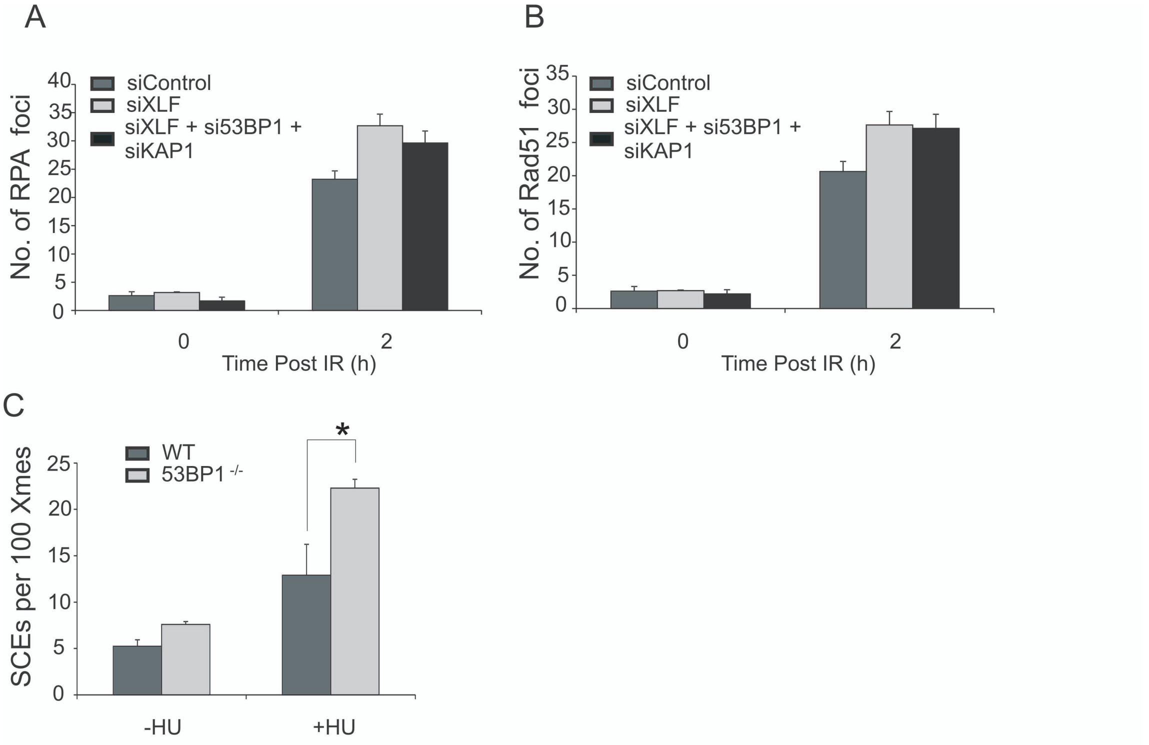

D

Target gene (Human)	Target sequence (5'-3')
BRCA2	GAAACGGACUUGCUAU UUA<br>GGUAUCAGAUGCUUCAUUA<br>GAAG AAUGCAGGUUUAAUA<br>UAAGGAACGUCAAGAGAUA
BRCA1	CAACAUGCCCACAGAUCAA<br>CCAAAGCGAGCAAGAGAAU<br>UGAUAAAGCUCCAGCAGGA<br>GAAGGAGCUUUCAUCAUUC
53BP1	GAAGGACGGAGUACUAAUA<br>GCUAUAUCCUUGAAGAUUU<br>GAGCUGGGAAGUAUAAAUU<br>GGACUCCAGUGUUGUCAUU
DNAPK-cs	GGAAGAAGCUCAUUUGAUU<br>GAGCAUCACUUGCCUUUAA<br>GCAGGACCGUGCAAGGUUA<br>AGAUAGAGCUGCUAAAUGU
Ku80	AAACUUCCGUGUUCUAGUG<br>GAGCAGCGCUUUAACAACU<br>CGAGUAACCAGCUCAUAAA<br>GCAUGGAUGUGAUUCAACA
XLF	GAGGGAGCUAGCAACGUUA<br>GGGCUACGCUGAUUCGAGA<br>CCUUCAGAUUCUUCGUAAA<br>AGAAAGAGUCCACGGGUAC

Figure S4

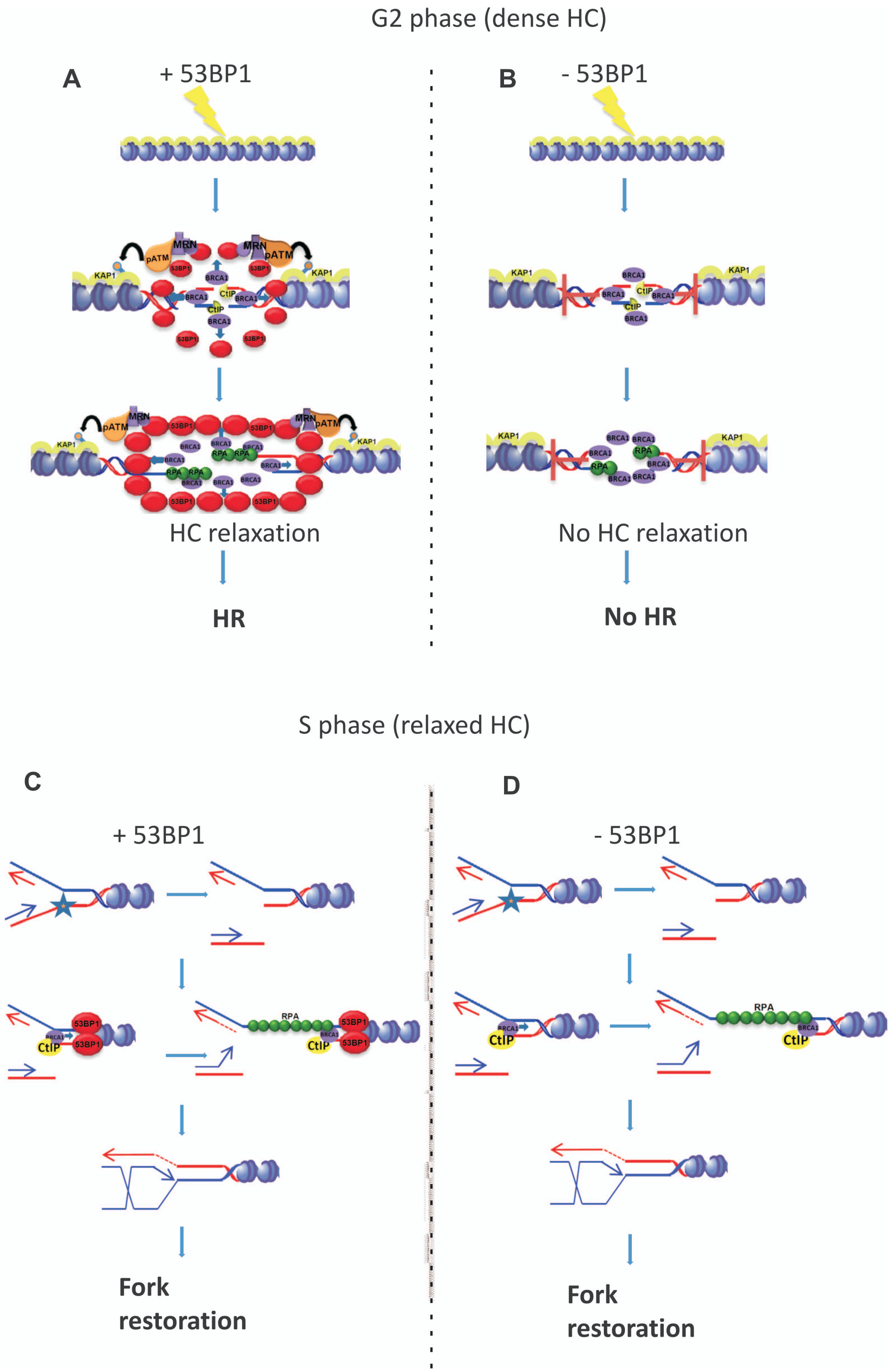
G2 phase (dense HC)
A
+ 53BP1
B
- 53BP1
MRN
pATM
KAP1
53BP1
BRCA1
CtIP
RPA
HC relaxation
HR
No HC relaxation
No HR
S phase (relaxed HC)
C
+ 53BP1
D
- 53BP1
Fork restoration
Fork restoration

Figure S5
